# Effect modification in the temperature extremes by mortality subgroups among the tropical cities of the Philippines

**DOI:** 10.3402/gha.v9.31500

**Published:** 2016-06-28

**Authors:** Xerxes T. Seposo, Tran Ngoc Dang, Yasushi Honda

**Affiliations:** 1 Graduate School of Comprehensive Human Sciences, University of Tsukuba, Tsukuba, Japan; 2 Department of Environmental Health, Faculty of Public Health, University of Medicine and Pharmacy, Ho Chi Minh, Vietnam; 3 Faculty of Health and Sports Sciences, University of Tsukuba, Tsukuba, Japan

**Keywords:** effect modification, mortality subgroups, tropicl cities, temperature–mortality, Philippines

## Abstract

**Background:**

Temperature–mortality relationships have been extensively probed with varying temperature range but with relatively similar patterns and in some instances are being modified by specific mortality groups such as causes of mortality, sex, and age.

**Objective:**

This study aimed to determine the risk attributions in the extreme temperatures and also identified the risks associated with the various mortality subgroups.

**Design:**

We used the 2006–2010 daily average meteorological and daily mortality variables from the Philippine Atmospheric Geophysical and Astronomical Services Administration and Philippine Statistics Authority–National Statistics Office, respectively. Mortality data were divided according to cause (cardiovascular and respiratory), sex, and age (0–14 years, 15–64 years, and >64 years). We performed a two-stage analysis to estimate the extreme temperature effects stratified by the different mortality subgroups to observe the effect modification.

**Results:**

In the pooled analysis, greater risks were observed in the extreme high temperature (99th temperature percentile; RR (relative risk)=2.48 CI: 1.55–3.98) compared to the extreme low temperature (1st temperature percentile; RR=1.23 CI: 0.88–1.72). Furthermore, effect modification by mortality subgroups was evident, especially higher risks for extreme temperatures with respiratory-related diseases, women, and elderly.

**Conclusions:**

Both sex and age were found to effect modify the risks in extreme temperatures of tropical cities; hence, health-related policies should take these risk variations into consideration to create strategies with respect to the risk population.

## Introduction

Climate action is one of the mainstream issues, which is now currently being addressed by various countries, signified through the commitment to the new Sustainable Development Goals (SDG) (1). With these new set of goals to be achieved by the end of 2030, combating climate change and its impact was explicitly singled out through the ‘*improvement of the institutional capacity on climate change mitigation, adaptation, impact reduction and early warning*’ (1, 2).

Although the provisions of the goals are made up by complex and intertwined dimensions of climate change action strategies, ultimately, the issue revolves around the notion of increasing temperature. This increase in temperature has also been linked to certain health risks, which has been observed to be a persistent and existing issue continuously being addressed via temperature–mortality trend identification and risk determination (3–5).

The effect of temperature and mortality has been extensively probed across the globe with varying temperature range but with relatively similar patterns (3, 6–8). Among the patterns observed, risks are evidently high in the extreme temperatures with considerably minimal risk in between, forming the occasionally observed U-, V-, and J-shaped patterns (9–11). All of these patterns have a minimum mortality temperature (MMT), otherwise known as optimum temperature, wherein risks associated with the said temperature are considered to be minimal when compared to mortality rates on days below or beyond (the minimum), thereby having the aforementioned patterns (12).

In some instances, the exposure–response relationship is being modified by sex and age, and also by causes of death, which eventually affects the relative risks (RR) (13, 14). Mortality, in this case, serves as the response variable to the varying exposures of temperature. Risks change along the temperature spectrum, with notably higher risks in the extreme temperatures, which sometimes lead to greater mortality with respect to the optimum temperature (15–17).

Even though extensive studies have evaluated the effect modification by mortality subgroups using the whole temperature range, only a few have explored the effect modification using the extreme temperature range (13, 17, 18). In our previous study (19), we have identified possible effect modification by age using the whole temperature range. Similar observations were also recorded, which highlights that cause of death, sex, and age have variations in the risks in the temperature extremes in various cities in the world (14, 20–23).

McMichael et al. (4) note that low- and middle-income countries, such as the Philippines (24), are prone to health vulnerabilities, which are caused by the changing climate. It is therefore necessary to enhance the profiling of the risk attributions, in order to equip the country and related low- and middle-income countries with relatively similar temperature range, with the necessary information to materialize precautions that can address the risks brought forth by the adverse temperature scenarios. Moreover, identification of risks with respect to the mortality subgroups will be essential components for risk population-tailored strategies. This study will explore how causes of mortality, sex, and age effect modify the temperature–mortality relationship in the extreme temperatures.

## Methods

### Study sites

**Fig. 1:**
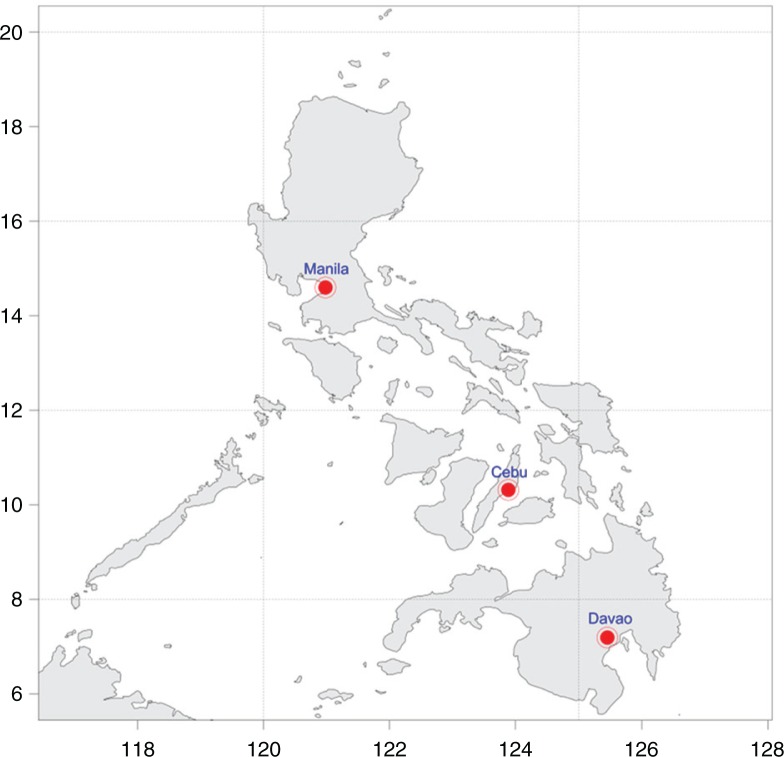
Geographical location of the three metropolitan cities in the Philippines.

The Philippines consists of 7,107 islands that can be clustered into three locally known big islands/clusters of Luzon, Visayas, and Mindanao. These three big clusters house the three metropolitan cities of the country, which serve as the centers of business and commerce in the respective cluster (Fig. 1).

### Meteorological and mortality data

We collected both the 2006–2010 daily average meteorological and daily mortality variables from the Philippine Atmospheric Geophysical and Astronomical Services Administration (PAGASA) and Philippine Statistics Authority–National Statistics Office (PSA-NSO), respectively. Mortality data were then divided according to cause of death, sex, and age. We used the ICD 10 codes to segregate cardiovascular-related deaths (I00–I99) and respiratory-related deaths (J00–J99) from the all-cause mortality counts, and we created three groups for age-specific mortality: 0–14 years, 15–64 years, and >64 years. We set the extreme low temperatures at the 1st and 5th temperature percentiles, and the extreme high temperatures at the 95th and 99th temperature percentiles.

### Statistical analyses

We performed a two-stage analysis to estimate the extreme temperature effects stratified by mortality subgroups to observe the effect modification. In the first-stage analysis, we analyzed the temperature–mortality relationship using a time series analysis with Poisson distribution, accounting for over-dispersion, subjected to a distributed lag nonlinear model (DLNM) parameterization, as shown below (25–27):

1 $$\log\left(\mu_{t}^{c}\right)=\alpha +\beta T_{t,l}+ns\left(date,7\times 5\right)+ns\left(RHave_{t},3\right)+as.factor\left(dow\right)+\varepsilon_{t}$$

where $\log\left(\mu_{t}^{c}\right)$ is the expected value of the log of mortality on city (*c*) and time (*t*); *α* is the intercept; *β* is the vector of regression coefficients for the cross-basis (*T*_*t,l*_) in predetermined temperature and lag dimensions; *ns* is the smoothing parameter set to natural cubic spline (NCS); *date* controls for seasonal variations with a total of 35 degrees of freedom (df); *RHave*_*t*_ is the relative humidity as a covariate on time (*t*) with 3 df. *dow* is the day of the week as a factor of categorical variables; *ɛ*_*t*_ is the residual. The selection of df for the covariates is based on previous studies (12, 26). In the model fitting process, we used the NCS specification in the cross-basis function of DLNM (19). By using the Quasi-Poisson Akaike Information Criterion (QAIC) for model parameterization (28), we were able to determine that the combination of 4 df for both temperature and lag dimensions, respectively, was considered to be the best fit having the least QAIC value.

In the second-stage analysis, we pooled the city-specific estimates using a random-effects meta-analysis:

2 $$\log\left(\mu_{t}^{c*}\right)=\hat{\beta }+\delta_{c}+\varepsilon_{c}$$

where $\log\left(\mu_{t}^{c*}\right)$ is the effects estimate of city (*c*) in the first-stage analysis, $\hat{\beta }$ is the pooled estimate to be determined with *δ*_*c*_ as a vector of within-city random effects by city (*c*), and *ɛ*_*c*_ represents the between-cities random errors (29, 30). City-specific estimates in the second-stage analysis were assumed to be normally distributed. After pooling the city-specific estimates, we stratified the pooled pattern by cause of death, sex, and age to determine the effects estimates due to effect modification.

All analyses were carried using R programming through the following packages: “*ggmap*” and “*maps*” for geographical location determination, “*dlnm*” for city-specific estimates estimation, and “*mvmeta*” for meta-analysis.

## Results

Table 1 shows the descriptive statistics of both meteorological and mortality data from the three cities in 2006–2010 with a total of 182,908 mortality counts and an average temperature well within the range at 28°C. Among the cities, 50% of the mortality counts were from Manila (*n*=94,656), with the other half from both Cebu and Davao. In order to observe the effect modification, we stratified by mortality subgroups, namely cause of death, sex, and age.

**Table 1:** Descriptive statistics of the meteorological and mortality statistics per city (*N*=182,908)

Variables (mean±SD)	Manila (*n*=94,656)	Cebu (*n*=43,830)	Davao (*n*=44,422)
Average temperature (°C)	28.8±1.52	28.2±1.16	28.1±1.00
Average humidity	73.9±7.46	82.5±5.43	82.1±4.35
All-cause mortality	52±8	24±5	24±5
Cause-specific mortality			
Cardiovascular	15±4	7±3	9±3
Respiratory	6±3	3±2	2±2
Sex-specific mortality			
Women	22±5	10±5	10±3
Men	30±6	14±6	15±4
Age-specific mortality			
0–14 years	9±3	3±2	2±1
15–64 years	27±6	12±9	13±4
> 64 years	17±4	9±3	10±3

**Fig. 2:**
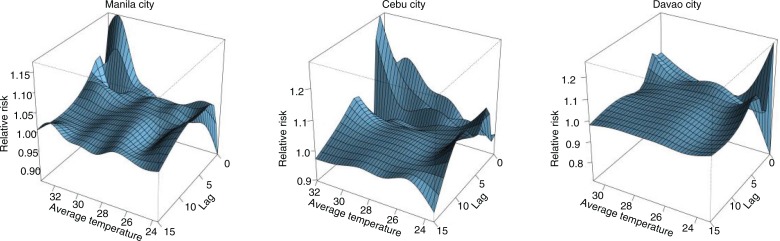
Distributed lag nonlinear relationship of average temperature, lag, and RR in the three cities from 2006 to 2010.

**Fig. 3:**
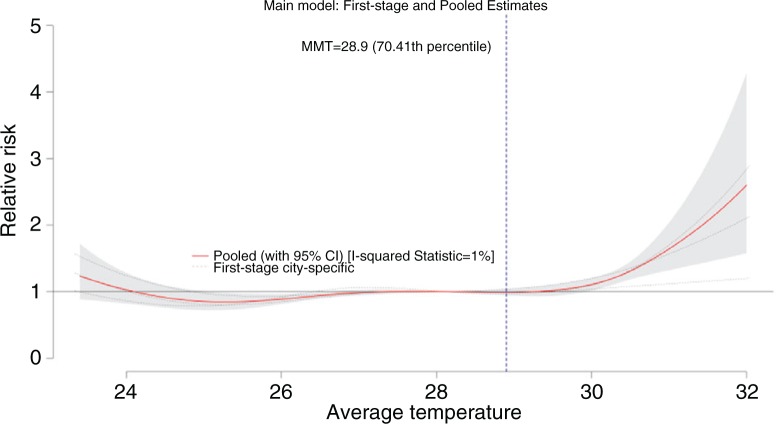
Meta-analysis of the pooled effects estimates from the three cities. The vertical blue marker serves as the point of MMT. The red line is the pooled estimate, while the dotted lines are the city-specific estimates (of the first-stage analysis).

Figure 2 shows the three-dimensional relationship of average temperature and RR on the various lags. All three cities have common immediate high-temperature effects in lags 0–2, with heightened risks observed in the lower temperature percentiles as shown in Supplementary Fig. 1. The effect estimates derived from the city-specific analysis through DLNM were then pooled via meta-analytical techniques as mentioned in the previous section and are shown in Fig. 3.

The red line, pooled estimate, which passes through the dotted lines, city-specific estimates, attempts to create a suitable fit with respect to the city-specific information/estimates. Because there is no monotonous rise in the RR, we used the second local minimum as a reference temperature, which, in this case, is also the MMT located at the 70th temperature percentile marked with a vertical blue line. The observed similarity in the all-cause mortality trend of the exposure–response relationship graphs among the three cities shown in Supplementary Fig. 2 resulted to a homogeneous pattern with an I-squared statistic equal to 1%. Evident high-temperature effects were observed in the pooled relationship at the 99th temperature percentile (RR=2.48 CI: 1.55–3.98) and an elevated risk in the 1st temperature percentile (RR=1.23 CI: 0.88–1.72).

**Fig. 4:**
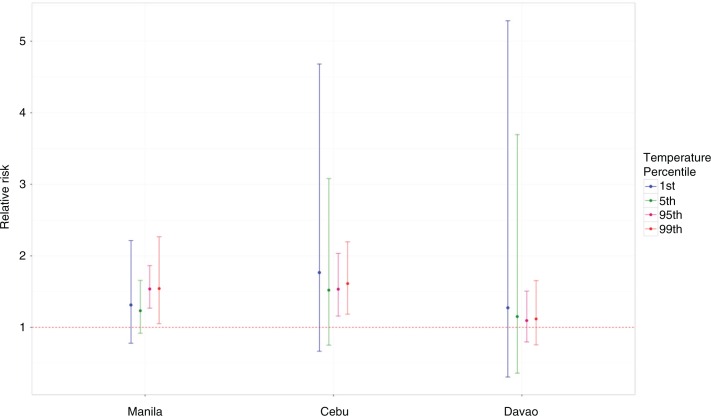
All-cause mortality per city on relative scale at the 1st, 5th, 95th, and 99th temperature percentiles.

**Fig. 5:**
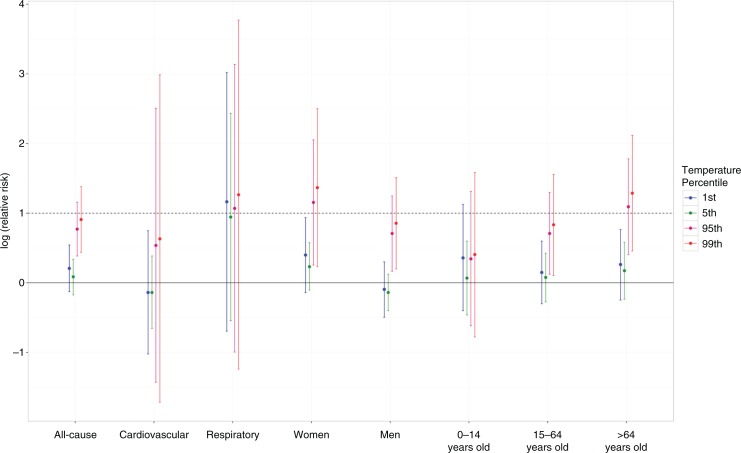
Log-transformed RRs showing the effect modification in the pooled pattern by various mortality subgroups at the 1st, 5th, 95th, and 99th temperature percentiles. The dotted line marks the log of RR at 1.

In Fig. 4, lower temperature effects were observed to be higher in Cebu and Davao, whereas Manila has higher risks in the higher temperature. For city-specific analysis, we used the relative scales, which may prove to have more relevant implications to city-specific attributes compared to using absolute scales (31). However, all-cause mortality might not truly reflect the trends by the mortality subgroups, and thus, inherent effect modification can be observed, as shown in Fig. 5.

We opted to log-transform the RRs in Fig. 5 because the non-transformed RRs had wide confidence intervals, which masked the other lower RRs. Among the mortality subgroups, we have observed variations in the causes of mortality in Supplementary Fig. 3.

In summary, the results have shown that at the aggregate level, using all-cause mortality, all the cities were found to be homogeneous. However, when disaggregated into the mortality subgroups, effect modification by cause of death, sex, and age was evident.

## Discussion

In this study, we explored the effect modification brought about by the different mortality subgroups in the extreme temperatures among the three cities in the Philippines. The main findings of the study are that 1) extreme high temperatures have greater risks compared to the different temperature percentiles; 2) higher risks were particularly observed in respiratory-related cases, women, and people aged >64 years; and 3) city-level variations in the risks can be linked to area-specific attributes.

The results of the study indicating that extreme high temperatures pose greater risks compared to the other parts of the temperature percentile are consistent with previous studies (4, 14, 17). A J-shaped pattern signifying an increased risk in the extreme high temperature is evident in the pooled pattern in Fig. 3 and can be clearly deciphered through the stratification by mortality subgroups in Fig. 5. Anderson and Bell (31) and other similar observations from other studies (8, 20) have shown that heat-related mortality, specifically in the extreme high temperature, is usually associated with shorter lags, which were observed to last from lags 0–2 (as shown in Supplementary Fig. 1). Nevertheless, extreme low-temperature effects were also observed in Supplementary Fig. 4 per city and per mortality subgroup, which lasts longer (at lag 5) than the extreme high-temperature effects as shown in Supplementary Fig. 1.

More importantly, this study explored the effect modification brought about by cause of death, sex, and age in both pooled and city-specific extreme temperature percentiles: the 1st and 5th being the extreme low temperature, and the 95th and 99th being the extreme high temperature. In Fig. 5, extreme high-temperature effects are prominent in individuals who have respiratory-related problems, females, and people aged >64 years. Respiratory causes of mortality having greater risk especially in the high temperatures is supported by previous studies, which indicated that hot temperature can be deleterious to people with chronic respiratory diseases (32). Michelozzi et al. (33) point out the possibility of exacerbations of chronic obstructive pulmonary disease (COPD) in the hospital setting, and which were likely due to problems with excess heat dissipation through circulatory adjustment (34). On that same note, Michelozzi et al. (33) stressed that extreme temperatures increase the risk of those with COPD in developing pulmonary vascular resistance secondary to peripheral pooling of blood or hypovolemia.

With regard to women and men, both sexes have similar risk patterns with respect to extreme high temperatures having greater risks compared to extreme low temperatures. However, between the two, women have greater risks, in either extreme temperatures, compared to men, which is in concurrence with the results of previous studies (22, 32, 35, 36). On the other hand, some studies showed that either men have greater risks (22) or no difference at all (37, 38). Although some researchers have reported that the difference between the two may be attributed to socioeconomic factors and of geographical context (37, 39), the underlying factors and mechanisms resulting in these varying results across different areas warrant further investigation.

Results from the age-stratified analysis showed that the elderly, aged >64 years, were experiencing the greatest risks in the extreme high temperature. This is consistent with previous studies, which indicate that the thermoregulatory capacity of a person deteriorates as the body ages (32, 33, 40). Other socioeconomic factors as well as social isolation can also affect the susceptibility of the elderly population (32). Harlan (41) notes that socially isolated elderly tend to have increased vulnerability to temperature effects. However, we were not able to explore this possibility because of the lack of individual socioeconomic parameters. The study's results with regard to the susceptibility with the various subgroups have similarities with those observed by D'Ippoliti et al. (32) and Yang et al. (23), whereby both studies have found that older females who suffer from respiratory-related diseases have greater risks. In Supplementary Fig. 3, pooled patterns of each individual characteristic across three cities were observed to have variations, most especially in the patterns of cardiovascular and respiratory causes of mortality. However, because of the limited number of cities, we were not able to carry out a meta-regression with area-specific meta-predictors, as there is little or no variation among the three cities, as shown in Fig. 3.

The study is limited to the following: 1) number of cities and 2) lack of air pollution data. Although there is no gold standard with respect to the number of cities to be included in the study, the inclusion of more cities may increase the statistical power of the analysis, likewise, will enable clearer detection of variations with respect to the explanatory variables. Also, we were unable to acquire the daily air pollution data for the said period, as the Philippines is currently institutionalizing the detection of particulate matter monitoring in the country.

The study has shown that greater risks were likely to be observed in the extreme temperatures. Furthermore, effect modification by mortality subgroups can be observed, especially with respiratory-related diseases, women, and elderly. Variations were observed in the causes of mortality; however, definite patterns are yet to be ascertained because of the limited number of cities included in the study. Generalizations of this study toward other tropical cities, or even within the Philippines, should be taken into caution because the city-specific variables may vary from one area to the other.

## Conclusions

This study has shown that effect modification by mortality subgroups is evident in the extreme temperatures of tropical cities and that health-related policies should take these variations in the risks into consideration in order to create strategies with respect to the population at risk.

## Supplementary Material

Effect modification in the temperature extremes by mortality subgroups among the tropical cities of the Philippines

## Paper context

The relationship of temperature and mortality had been extensively studied and has a consistent non-linear pattern among cities across the globe, with the relationship being effect modified by mortality subgroups focusing on the whole temperature range. This study explored effect modification in extreme temperatures by different subgroups. Effect modification by respiratory-related diseases, women, and elderly mortality subgroups was observed. Health-related policies should take these risk variations into account to engineer strategies for the risk populations.
