# Supplementary material for: Effect modification in the temperature extremes by mortality subgroups among the tropical cities of the Philippines
Source: Glob Health Action. 2016 Jun 28;9:10.3402/gha.v9.31500. doi: 10.3402/gha.v9.31500 (PMC4928071; doi:10.3402/gha.v9.31500)
Supplement: Effect modification in the temperature extremes by mortality subgroups among the tropical cities of the Philippines [file GHA-9-31500-s001.docx]

Supplementary Materials

**Effect Modification in the Temperature Extremes by Individual Characteristics in Tropical Cities of the Philippines**


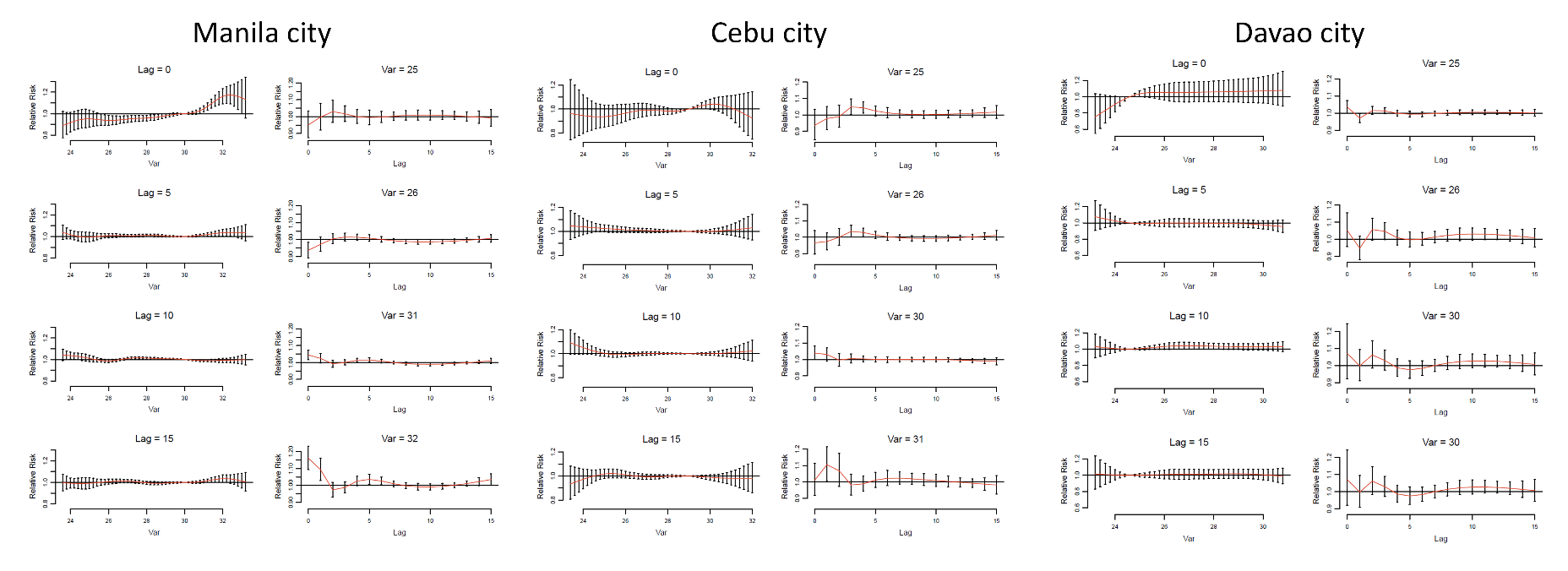


Figure S1. Dose-response slices of the three metropolitan cities on their respective lag and temperature dimensions


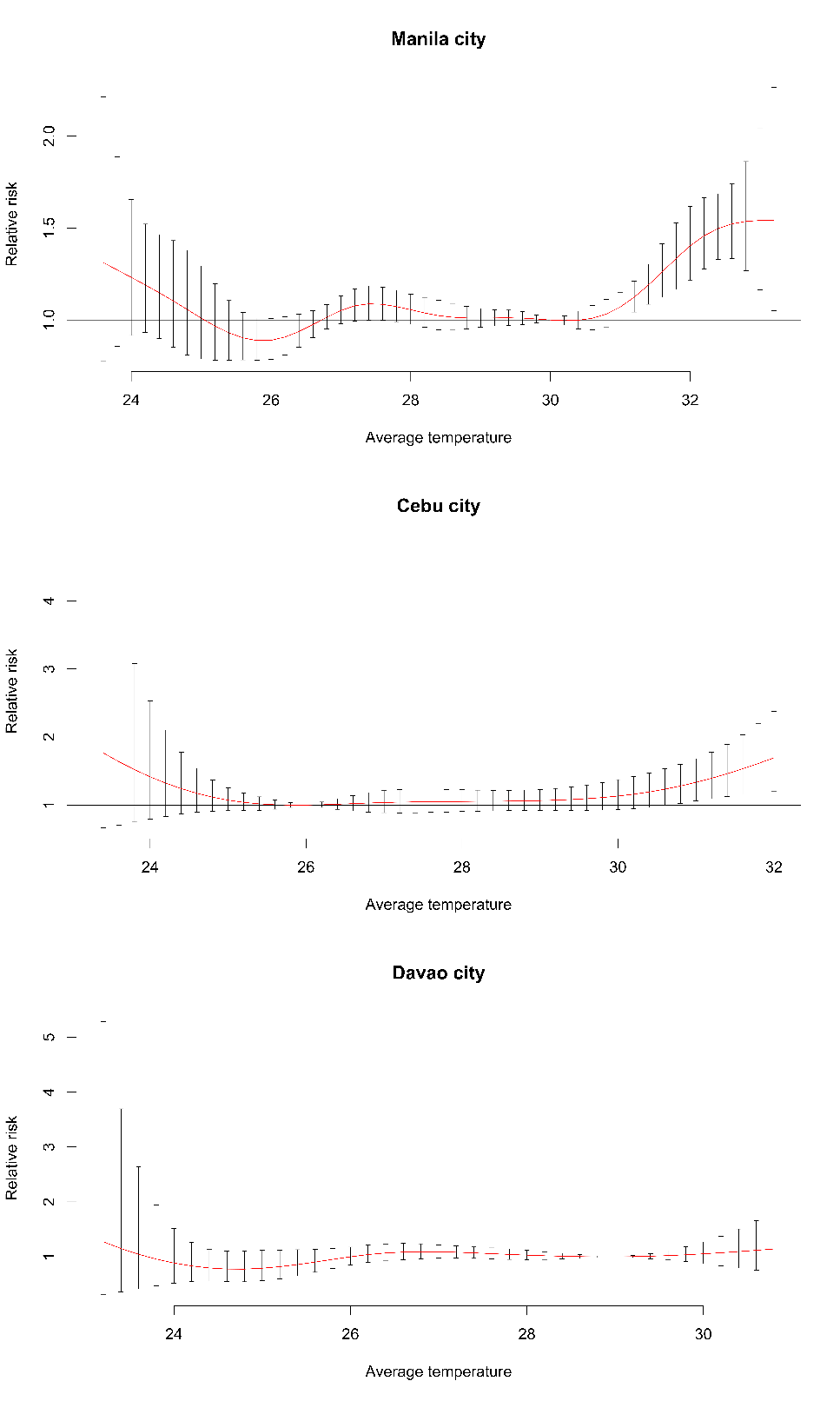


Figure S2. Overall dose-response patterns of the three metropolitan cities


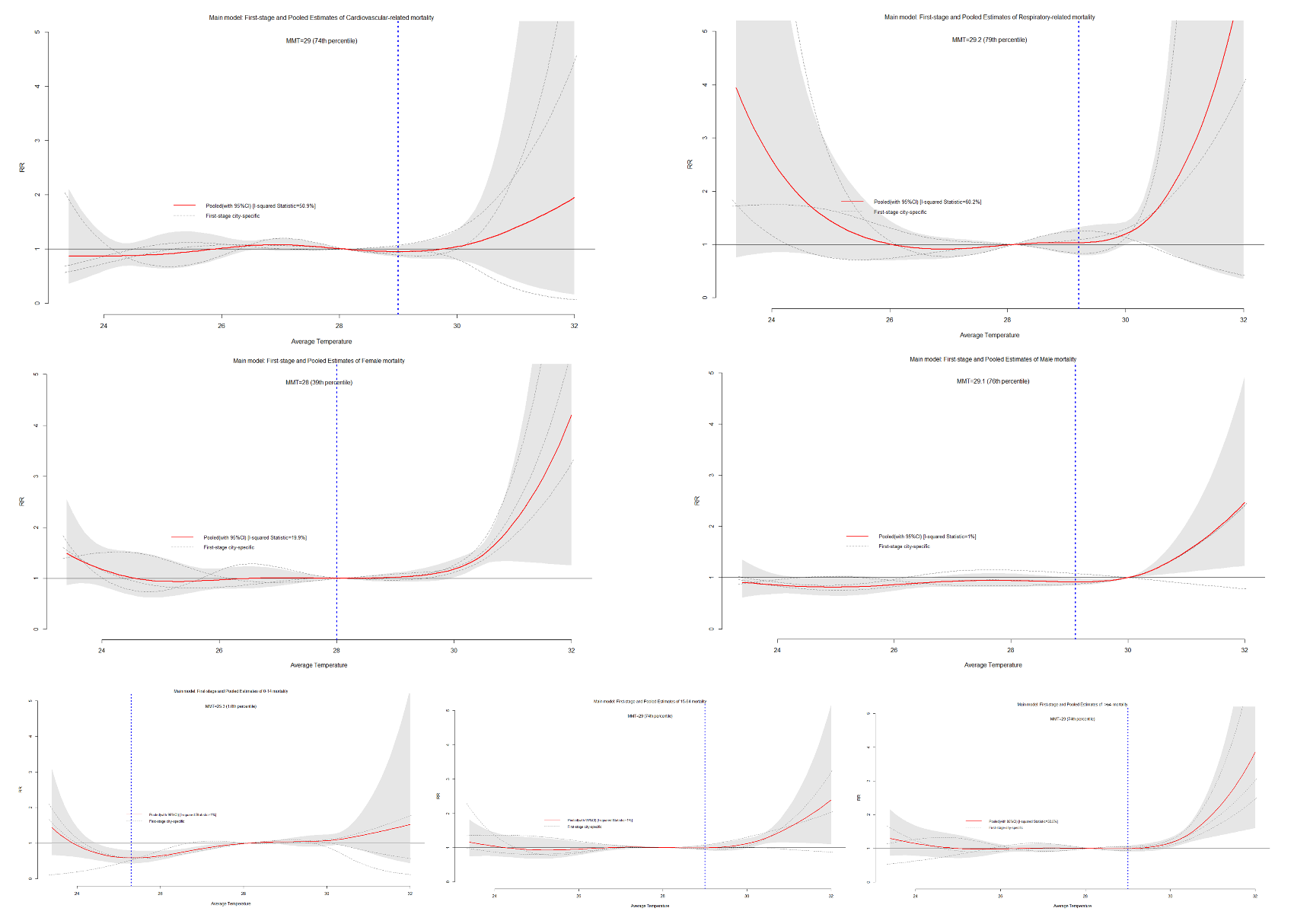


Figure S3. Meta-analytic graphs and the pooled patterns of the individual characteristics. The jagged lines are the city-specific temperature-mortality patterns, the red solid line is the pooled pattern, and the jagged, blue vertical line is the MMT point.


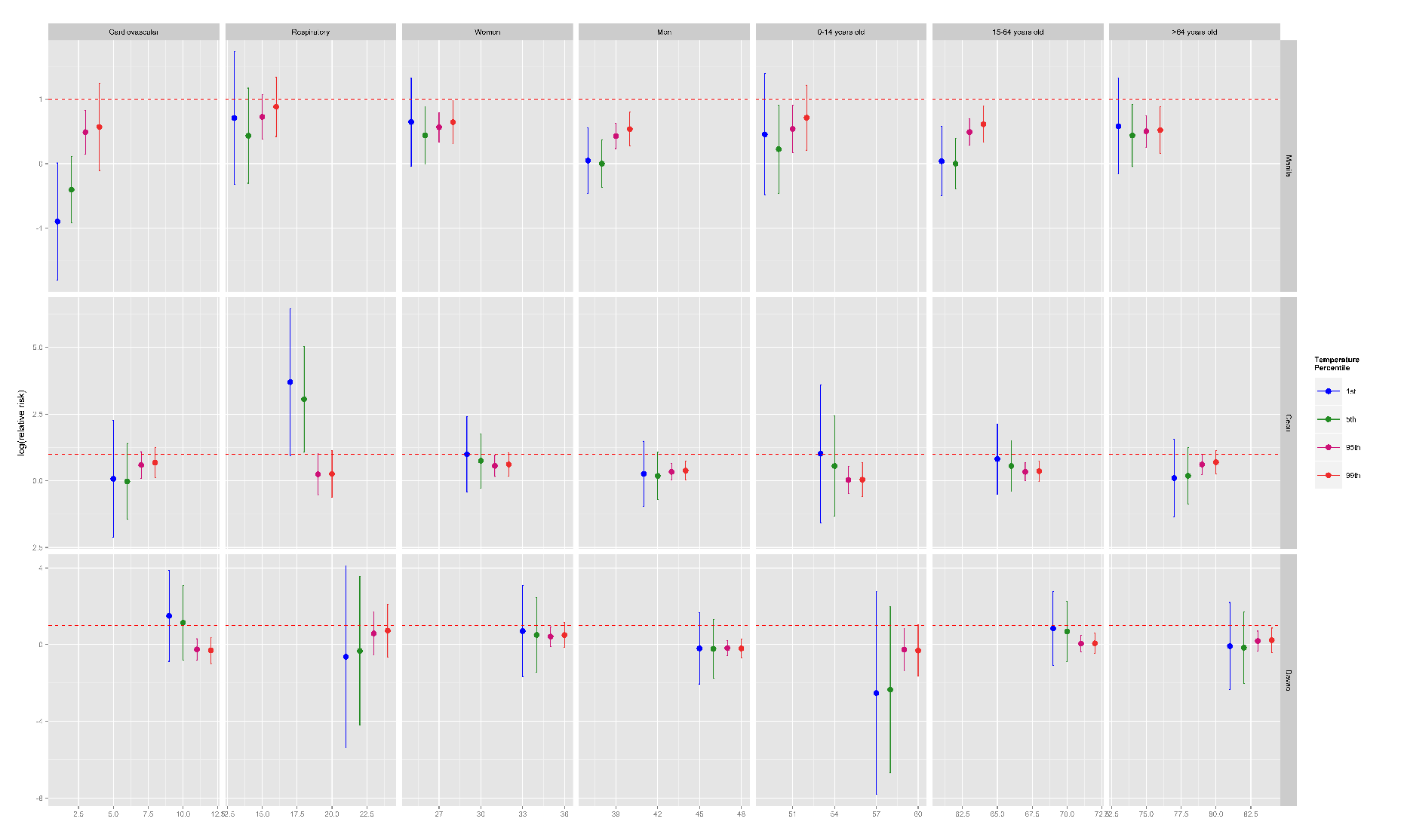


Figure S4. Individual- and city-specific log of the RR on the 1^st^, 5^th^, 95^th^, and 99^th^ temperature percentiles. (We allowed free-dimensionality of the scales with respect to the temperature and log of the RR due to extremely varied RRs which masks the lower RRs.)
